# A pair of datasets for microRNA expression profiling to examine the use of careful study design for assigning arrays to samples

**DOI:** 10.1038/sdata.2018.84

**Published:** 2018-05-15

**Authors:** Li-Xuan Qin, Huei-Chung Huang, Liliana Villafania, Magali Cavatore, Narciso Olvera, Douglas A. Levine

**Affiliations:** 1 Department of Epidemiology and Biostatistics, Memorial Sloan Kettering Cancer Center, New York, NY, USA; 2 Marie-Josée & Henry R. Kravis Center for Molecular Oncology, Memorial Sloan Kettering Cancer Center, New York, NY, USA; 3 Department of Surgery, Memorial Sloan Kettering Cancer Center, New York, NY, USA

**Keywords:** Data processing, miRNAs, Microarrays

## Abstract

We set out to demonstrate the logistic feasibility of careful experimental design for microarray studies and its level of scientific benefits for improving the accuracy and reproducibility of data inference. Towards this end, we conducted a study of microRNA expression using endometrioid endometrial tumours (*n*=96) and serous ovarian tumours (*n*=96) that were primary, untreated, and collected from 2000 to 2012 at Memorial Sloan Kettering Cancer Center. The same set of tumour tissue samples were profiled twice using the Agilent microRNA microarrays: once under an ideal experimental condition with balanced array-to-sample allocation and uniform handling; a second time by mimicking typical practice, with arrays assigned in the order of sample collection and processed by two technicians in multiple batches. This paper provides a detailed description of the generation and validation of this unique dataset pair so that the research community can re-use it to investigate other statistical questions regarding microarray study design and data analysis, and to address biological questions on the relevance of microRNA expression in gynaecologic cancer.

## Background & Summary

Genomic profiling of molecular features such as gene expression levels entails a complex multi-stage experiment^1^. Systematic variations in experimental handling factors, such as lab technicians and image scanners, can lead to undesirable variations in the data that increase data variability and confound the biological signal of interest^2,3^. A typical practice for combating such unwanted variations is to use post-hoc data adjustments such as normalization^4–6^. An alternative to post-hoc adjustment is to carefully design the experiment, using time-tested statistical principles such as blocking and randomization, to balance handling effects between sample groups of interest and abate their negative impacts on data inference^7–9^. However, such careful design has received little attention in genomic studies, possibly due to lack of awareness and the perceived level of logistic difficulty in implementing them.

We aim to demonstrate that (1) careful experimental design is logistically feasible in clinical microarray studies and (2) it can provide significant scientific benefits that warrant the planning effort. Towards this end, we conducted a study of microRNA expression in endometrioid endometrial tumours (*n*=96) and serous ovarian tumours (*n*=96) that were primary, untreated, and collected during 2000–2012 at Memorial Sloan Kettering Cancer Center. The same set of tumour tissue samples was profiled twice using the Agilent microRNA microarrays: once under an ideal experimental condition with balanced array-to-sample-group allocation (via the use of blocked randomization) and uniform handling (by an experienced technician in a single processing run); a second time by mimicking typical practice with arrays assigned to samples in the order of sample collection and processed by two technicians in multiple batches. Differential expression between the two sample groups was assessed in the uniformly-handled dataset to serve as a benchmark; it was also assessed in the non-uniformly-handled dataset, both before and after normalization, and compared with the benchmark. Additional datasets were simulated by estimating (1) biological effects for the samples (serving as ‘virtual samples’) and (2) handling effects for the non-uniformly-handled arrays (serving as ‘virtual arrays’) from the paired datasets, and then re-allocating and re-hybridizing the virtual arrays to the virtual samples following various configurations of blocking, stratification, and randomization in the presence of handling effects^7^.

In this paper we provide a detailed description of the generation and validation of this unique pair of datasets, so that the data can be re-used by the research community to investigate additional statistical questions regarding experimental design and data analysis for microarray studies and to address biological questions on microRNA expression in gynaecologic tumours. We note that the experiments in our study were not designed specifically for gynaecologic tumour samples, and our approach of the paired datasets can be used for samples of other tissue types as well.

## Methods

All human tumour tissues used in this study were obtained from participants who provided informed consent and their use in our study was approved by the Memorial Sloan Kettering Cancer Center Institutional Review Board.

### Sample collection

Ninety-six endometrioid endometrial tumour samples and 96 serous ovarian tumour samples were used in our study. All tumour samples were primary, previously untreated, and collected during 2000–2012 at Memorial Sloan Kettering Cancer Center.

### RNA extraction

All sample preparation followed strict quality control standards to ensure that RNA extraction was as uniform as possible. Once tissue was harvested, it was snap frozen for cryomold embedding. A 5-μm histologic section was cut from the top of the cryomold to evaluate the content and percentage of necrosis. Specimens with less than 60% tumour cell nuclei had gone through macro-dissection aiming to remove non-tumour sections to further enrich the specimen. In this study, all specimens had less than 20% necrosis. A gynecologic pathologist evaluated all specimens to identify histologic cell type, malignancy grade and site of origin. Ambion mirVana microRNA Isolation Kit was used to extract RNA from 30 to 100 mg of macro-dissected cryomold tissue. Total RNA yield and quality were assessed using the NanoDrop spectrophotometer and the Agilent Bioanalyzer. All slides were cut by a senior histotechnologist and all RNAs were extracted by an experienced technician. RNAs from the same aliquot were used for the two arrays of the same tumor sample.

### Microarray data generation

The extracted RNAs were profiled for microRNA expression using the Agilent Human microRNA microarray v16.0, which contained 3,523 markers representing 1,205 human and 142 human viral microRNAs (Agilent Technologies, Sana Clara, CA). Fluorescence labelling of the extracted RNAs, hybridization to the arrays, slide washing, and image scanning all followed the manufacturer's instructions. Image data were extracted using Feature Extraction 10.7.3.1 (Agilent). Arrays used for the first study (with careful design) were ordered from the same manufacture batch, and arrays used for the second study were ordered from two separate manufacture batches.

### Experimental design of the paired studies

In the first study, arrays were assigned to tumour samples using blocked randomization and were processed by an experienced technician in a single run. The data from this study is referred to as Data Citation 1.

− Blocking is the assigning of experimental units in each block of units to sample groups in proportion to group sizes^10^. Agilent microRNA microarrays come in 8-plex slides (with 8 arrays on each slide), which serve as blocks. In this study, 24 slides containing 192 arrays were used for the 192 tumour samples, with four arrays on each slide assigned to each sample group. Blocking can balance handling effects between two (pre-specified) sample groups so that they can cancel out in the analysis of differential expression comparing the two groups.
− On each slide eight arrays are arranged in two rows and four columns. In order to avoid any positional effect on the slide, array assignment was further stratified by slide row and column, with equal numbers of arrays on each row and each column assigned to the two sample groups. For a 2 by 4 array slide, there are a total of six possible configurations that allow row and column balance.
− Randomization is the assignment of arrays to samples in a random manner^10^. It can likely balance handling effects, with the level of likelihood positively correlated with the sample size. It is particularly useful when the primary outcome of interest is unknown or when there are secondary outcomes of interest.
− Randomization, in combination with blocking, is the allocation of arrays to sample groups with blocking first, and then assigning the arrays allocated to a sample group to samples in that group in a random manner.
− When implementing the array assignment for our study, we randomly assigned the 24 slides to four repetitions of the six row-column-balanced configurations, and then randomly assigned arrays allocated to a sample group to tumor samples in that group^7^.

In the second study, arrays were assigned to tumour samples in the order of sample collection and were handled by two technicians in five batches (with each batch on a separate date). More specifically, two batches of 40 arrays each were handled by one technician (the same technician who handled the first study), and three batches of size 34, 38, and 40 were handled by another technician. The data from this study is referred to as Data Citation 2.

### Data pre-processing

Data pre-processing for Data Citation 1 (which resulted from careful study design) included two steps: (1) log2 transformation, and (2) marker-replicate summarization using the median^7^. The Agilent microRNA array platform includes 10 to 40 replicates for each of the 3,523 markers. The between-replicate variation was very small in our data, which allowed us to use a simple median to summarize the replicates for each marker^7^.

Data pre-processing for Data Citation 2 included three steps^11^: (1) log2 transformation, (2) data normalization using quantile normalization, and (3) marker-replicate summarization using the median. We focus on the use of quantile normalization for the normalization step in this paper, and refer the readers to our previous publication for the use of other normalization methods^7^.

In addition to the 3,523 markers representing microRNAs, 7 negative control markers and 37 positive control markers were included on the Agilent microRNA array. The data for these control markers were included in Gene Expression Omnibus (GEO) database submission of the two datasets.

### Code availability

Data reading, pre-processing, and analysis were done in *R 3.2.3*. Codes for reading the raw Agilent data files into in *R* (Supplementary File 1, Supplementary File 2), and for pre-processing the data and comparing the data between the two sample groups to assess differential expression (Supplementary File 3) are available in the Supplementary Materials.

## Data Records

Microarray data are available in GEO: Data Citation 1 and Data Citation 2. Each data entry contains the raw Agilent data files and the pre-processed data matrix for microRNA expression, as well as the tumour type variable and the array batch variable. A SuperSeries record (GSE109059) is also available to provide access to both datasets.

## Technical Validation

### Quality check of extracted RNAs

**Figure 1:**
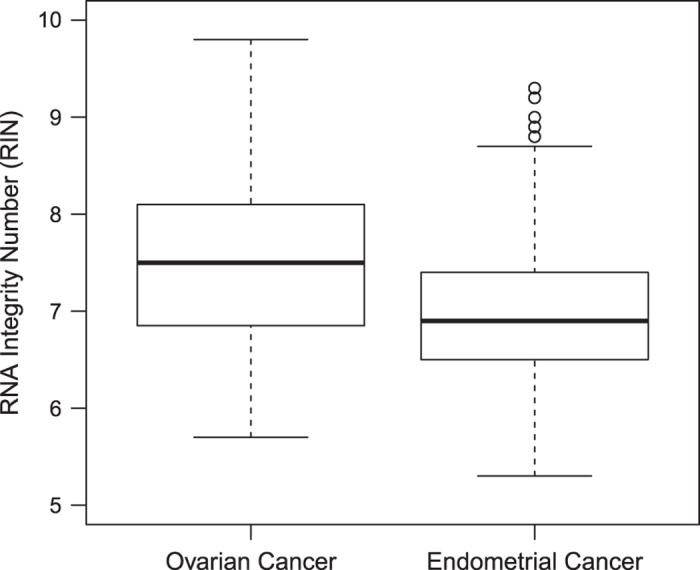
Boxplot of the RNA Integrity Number for the extracted RNAs used in our study. The left box is for the 96 ovarian tumour samples, and the right box is for the 96 endometrial tumour samples.

**Figure 2:**
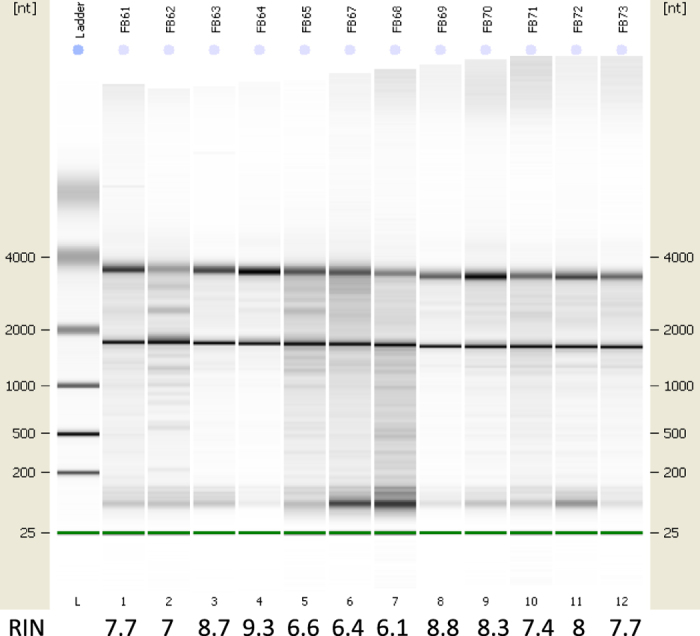
Gel image of the extracted RNAs for 12 of the tumour samples.

Extracted RNAs of tumour samples were checked on their quality based on the RNA Integrity Number (RIN) (Fig. 1) and the gel image pattern (Fig. 2). Only those that were of satisfactory quality were used in our study.

## Additional information

**How to cite this article**: Qin, L.-X. *et al.* A pair of microarray datasets for microRNA expression profiling to examine the use of careful study design for assigning arrays to samples. *Sci. Data* 5:180084 doi: 10.1084/sdata.2018.84(2018).

**Publisher’s note**: Springer Nature remains neutral with regard to jurisdictional claims in published maps and institutional affiliations.

## Supplementary Material



Supplementary File 1

Supplementary File 2

Supplementary File 3

## References

[d1] Gene Expression Omnibus 2018 GSE108838

[d2] Gene Expression Omnibus 2018 GSE109058

